# Mechanical energy and propulsion mechanics in roller-skiing double-poling at increasing speeds

**DOI:** 10.1371/journal.pone.0255202

**Published:** 2021-07-28

**Authors:** Jørgen Danielsen, Øyvind Sandbakk, David McGhie, Gertjan Ettema

**Affiliations:** Centre for Elite Sports Research, Department of Neuromedicine and Movement Science, Faculty of Medicine and Health Sciences, Norwegian University of Science and Technology, Trondheim, Norway; Universita degli Studi di Verona, ITALY

## Abstract

**Objectives:**

The aim of this study was to examine the effect of speed on mechanical energy fluctuations and propulsion mechanics in the double-poling (DP) technique of cross-country skiing.

**Methods:**

Kinematics and dynamics were acquired while fourteen male skiers performed roller-skiing DP on a treadmill at increasing speeds (15, 21 and 27 km∙h^-1^). Kinetic (E_kin_), potential (E_pot_), and total (E_body_) body mechanical energy and pole power (P_pole_) were calculated. Inverse dynamics was used to calculate arm power (P_arm_). Trunk+leg power (P_T+L_) was estimated, as was the power associated with body movements perpendicular to goal-direction (${\dot{\text{E}}}_{{\text{body}}_{⊥}}$).

**Results:**

E_kin_ and E_pot_ fluctuated out-of-phase throughout the cycle, at first sight indicating that pendulum-like behaviour occurs partly in DP. However, during the swing phase, the increase in E_pot_ (body heightening) was mainly driven by positive P_T+L_, while the decrease in E_kin_ was lost to rolling friction, and during the poling phase, considerable positive P_arm_ generation occurs. Thus, possible exchange between E_kin_ and E_pot_ seem not to occur as directly and passively as in classic pendulum locomotion (walking). During the poling phase, ${\dot{\text{E}}}_{{\text{body}}_{⊥}}$fluctuated out-of-phase with P_pole_, indicating a transfer of body energy to P_pole_. In this way, power generated by trunk+leg mainly during the swing phase (body heightening) can be used in the poling phase as pole power. At all speeds, negative P_T+L_ occurred during the poling phase, suggesting energy absorption of body energy not transferred to pole power. Thus, DP seem to resemble bouncing ball-like behaviour more than pendulum at faster speeds. Over the cycle, P_arm_ contribution to P_pole_ (external power) was 63% at 15 km∙h^-1^ and 66% at 21 and 27 km∙h^-1^, with the remainder being P_T+L_ contribution.

**Conclusions:**

When speed increases in level DP, both power production and absorption by trunk+leg actions increase considerably. This enhanced involvement of the legs at faster speeds is likely a prerequisite for effective generation of pole power at high speeds with very short poling times. However, the relative trunk+leg power contribution did not increase at the speeds studied here.

## Introduction

Double poling (DP) is an important cross-country skiing sub-technique mainly used on flatter parts of a course [1–3]. In DP, propulsive force and power can only be generated through the poles during the poling phase (pole-ground contact) while the skis continuously glide forward. Despite this, DP is a whole-body movement where leg involvement is essential for effective generation of these pole forces [4–6], with the legs being the main energy consumers (in absolute terms) [7]. In terms of mechanical energy and work, it has been estimated that the legs contribute approximately 30–40% of net mechanical power at low intensities, both in ergometer [8] and in uphill roller-skiing DP [9]. No studies have examined the power contribution from the arms and legs in (roller)-skiing DP on a level (flat) surface.

The way in which involvement of the legs can contribute in generating pole power can be described as follows. Considerable leg power is generated by leg extensions during the swing phase as the body and poles are repositioned for the next cycle. Here, the body is heightened, leading to an increase in body potential energy (E_pot_) [4, 6, 8, 10–12]. Towards the end of the swing and throughout most of the poling phase, E_pot_ decreases as the body is lowered and leaned forward [4, 6, 8]. A large part of this decreasing body energy can then be transferred and used in performing poling work (propelling centre of mass forward) [9, 11]. As such, mechanical work performed by the legs during swing are used to increase the amount of work performed on the poles during poling beyond what is possible if one was to rely more exclusively on arm work. For example, constraining the capability to use the legs (and trunk) lead to a (potentially substantial) reduction in the capacity to generate pole force and power [5, 13].

The (indirect) evidence for this energy exchange is based on a rationale being the out-of-phase fluctuations in body energy and external work (poling power) [11]. Also, the sum of upper extremity joint power does not equal net external power, meaning that the rest power must come from somewhere else [8]. With minimal option for the legs to contribute directly to poling power during the poling phase [8, 9, 11], the only energy source left that can contribute to doing poling work is body mechanical energy transfer. Thus, the repetitive heightening-and-lowering of the body and the associated large body energy fluctuations (especially E_pot_) can be considered as an essential characteristic of DP technique [5, 11]. This is especially the case when DP speed or intensity is to be increased, during which skiers rely heavily on increased involvement of the legs [4, 8, 10, 13, 14].

A main effect of increasing speed in level DP is a shortening of poling time (<0.2 s at ~30 km∙h^-1^), during which the ability to generate pole force and power can become a major challenge [4, 10]. Skiers then adopt to the more jumping-like DP technique, in which body mass is used to a larger extent to generate high pole force [1, 6, 10]. In mechanical energy terms, one would then expect an increase in leg power contribution. This hypothesis was confirmed in ergometer DP, where leg power contribution increased from ~40% at low to ~54% at near-maximal intensity [8]. However, in uphill DP at two different inclines, arm power contribution remained unaffected by speed, being ~63% and ~54% at moderate and steep inclines, respectively.

In that study [9], it was found at moderate incline that a large part of the decreasing body energy (during poling) was absorbed again by the legs rather than being transferred to pole power. The amount of energy absorption also increased much more at moderate than at steep incline when speed increased, meaning that more body energy apparently was transferred to pole power at the steep incline. That somewhat surprising finding was related to the effect of incline and thus different boundary conditions on movement execution [see 9]. Essentially, at steeper inclines, the poles can take more body weight than while at moderate inclines, more of the downward momentum generated by lowering the body must be counteracted by the legs, leading to greater energy absorption. However, it was argued there that the pronounced body heightening-and-lowering and associated positive-negative leg power at moderate incline still seemed necessary for effective generation of high arm (and pole) power in a relatively short amount of time [9]. Based on the findings at moderate and steep incline, it remains to be examined how pole force and power is generated in terms of origin of power (arms versus legs) and the effect of speed on these propulsion mechanics in roller-skiing DP on level (flat) terrain.

The aim of this study was to examine the effect of speed on mechanical energy fluctuations and propulsion mechanics in roller-skiing DP. First, it was expected that the repetitive heightening and lowering of the body and the associated body energy fluctuations would become more pronounced at faster speeds. Then, it was expected that fluctuations in the rate of change in body energy and poling power would be out-of-phase during poling. Such an out-of-phase fluctuation would indicate that body energy is transferred to pole power, i.e., power generated by the legs during the swing phase are used to generate pole power during the poling phase. One main question was whether the power contribution from the legs would increase (as in ergometer DP) or not (as in uphill DP) at faster speeds in level DP.

## Methods

### Participants

Fourteen male Norwegian XC skiers of national and international level (mean ± SD: age 23.7 ± 2.6 yrs, height 1.83 ± 0.05 m, body mass 76.1 ± 6.6 kg) voluntarily took part in this study. All participants were familiar with roller-skiing on a treadmill from their daily training and testing routines. All participants were informed about the nature of the study before providing written informed consent. The right to withdraw at any point was explicitly stated. The study was registered at and approved by the Norwegian Social Science Data Services, and the study was conducted in accordance with the Declaration of Helsinki.

### Experimental design

All skiers completed a warm-up for ~15 minutes consisting of DP roller-skiing on a treadmill at low and moderate speeds, with the inclusion of some short bouts at higher speeds. The same pair of roller skis were used by all skiers, wherein the 15-min warm-up period ensured the wheels and bearings reaching stable temperatures.

The main experiment consisted of DP roller-skiing at low (15 km∙h^-1^), moderate (21 km∙h^-1^), and high (27 km∙h^-1^) speeds on a treadmill. The treadmill incline was set at 1% increase work load somewhat (due to the lack of air resistance) and was otherwise considered level. The skiers were instructed to remain in approximately the same position on the treadmill and to keep self-selected cycle rates stable throughout the ~100 s trials in which kinetics and kinematics were recorded during the last ~75 s.

### Instruments and materials

Roller-skiing was performed on a 5 x 3 m motor driven treadmill (Forcelink Technology, Culemborg, The Netherlands). The same pair of classical roller skis with resistance category 2 were used by all skiers (IDT Sports, Lena, Norway). Poles of preferred length were available in 5 cm increments (Madshus UHM 100, Biri, Norway). The poles were instrumented with special carbine tips which ensured good grip on the treadmill belt surface covered with non-slip rubber. A safety harness connected to an emergency break secured all skiers. The coefficient of rolling resistance (μ) of the roller skis was determined three times before and after the experiments by a towing test as previously described [15]. The mean value of μ was the same before and after the experiments (0.018 ± 0.001).

### Kinetics

Axial ground reaction forces along the poles were recorded (1500 Hz) with CDF Miniature Button Load Cells (diameter, 15 mm; height, 8 mm; capacity, 2 kN; non-linearity, < .5%; weight, 10 g; Applied Measurements LTD, Aldermaston, Berkshire, UK) [16]. The load cells were placed on top of an aluminum tube (50 g), directly mounted at the top of and inside the pole tube. To minimize possible cross-talk between forces directed along the pole and forces related to squeezing, bending, or rotation of the hand grip, a small 8 mm diameter ball was located in between the load cell and the aluminum tube. Calibration of the pole forces were done by performing several poling like actions against a force platform (Kistler 9286BA, Kistler Instruments, Winterhur, Switzerland). The maximal error during peak force was about 10–15 N. Pole force data was amplified using a telemetric system (TeleMyo DTS, Noraxon, Scottsdale, AZ, USA) connected to a personal computer and the Qualisys Track Manager software (Qualisys AB, Gothenburg, Sweden).

### Kinematics

Nine infrared Oqus cameras (Oqus 400, Qualisys AB) captured three-dimensional position characteristics of passive reflective markers (ø 14 mm) at a sampling frequency of 250 Hz. Calibration of the measurement volume was done according to the manufacturer’s specifications. All markers were placed bilaterally on anatomical landmarks by the same researcher using double sided tape (3M, St. Paul, MN, USA). These landmarks were: on the ski boot corresponding to the head of the fifth metatarsal and to the lateral malleolus (ankle), the lateral femoral epicondyle (knee), the greater trochanter (hip), the lateral end of the acromion process (shoulder), the lateral humeral epicondyle (elbow), and the styloid process of ulna (wrist). These markers defined 11 body segments; feet, shanks, thighs, upper arms, forearms and trunk [e.g., 17]. One marker was placed ~5 cm below the grip handle of each pole and one marker on the lateral side of the pole tips. These markers defined the pole direction and thus the direction of pole forces. One marker was placed 1 cm behind the front wheel and one marker 1 cm in front of the back wheel on each ski.

### Data analysis

Kinetics and kinematics were synchronized and stored using the Qualisys Track Manager, and further analysis was performed in Matlab (R2016b, Mathworks Inc., Natick, MA, USA). Marker position and force data were low-pass filtered (8^th^ order, zero-lag, Butterworth filter) with the same cut-off frequency of 15 Hz [18, 19]. Masses, moments of inertia, and centre of mass of body segments were estimated according to de Leva [20]. Segment lengths were determined from the average of marker coordinates over the entire period of analysis. Centre of mass position of the forearms and trunk was adjusted to include the hands and head, respectively. Velocity and acceleration data were obtained by numerical differentiation with respect to time. The poling phase was defined as the period when the poles were in contact with the treadmill belt, that is, when magnitude and direction of velocity of the markers on the pole tips and treadmill belt were close to identical. The swing phase was defined as the period when the poles were off the belt.

To examine mechanical energy fluctuations, the kinetic energy of the body (E_kin_) was calculated as:

$${\text{E}}_{\text{kin}}=\sum_{i=1}^{11}\frac{1}{2}{\text{m}}_{\text{i}}{\text{v}}_{\text{i}}{}^{2}\text{+}\sum_{i=1}^{11}\frac{1}{2}{\text{I}}_{\text{i}}\omega_{\text{i}}{}^{2}$$ (1)

where m is mass (kg), v is the instantaneous absolute velocity in the coordinate system moving with treadmill belt speed, I is moment of inertia (kg·m^2^), and ω is angular velocity (rad·s^-1^) of the i^th^ segment.

Potential energy of the body (E_pot_) was calculated as:

$${\text{E}}_{\text{pot}}=\sum_{i=1}^{11}{\text{m}}_{\text{i}}{\text{gh}}_{\text{i}}$$ (2)

with h the instantaneous height of the i^th^ segment in the coordinate system moving with treadmill belt speed and g the gravitational acceleration (9.81 m∙s^-2^). Total body mechanical energy (E_body_) was calculated as the sum of E_kin_ and E_pot_ [17, 21]. In DP, the instantaneous (net) power output (P_o_) is used to induce changes in E_body_ and to overcome rolling resistance and gravity [9, 22] and was calculated as:

$${\text{P}}_{\text{o}}={\text{dE}}_{\text{body}}/{\text{dt}+\text{P}}_{\text{roll}}$$ (3)

where P_roll_ is the power against rolling resistance. P_roll_ was calculated as:

$${\text{P}}_{\text{roll}}=(\text{mg}\;\cos\;\alpha -{\text{F}}_{{\text{pole}}_{⊥}}){\mu \text{v}}_{{\text{ski}}_{∥}}$$ (4)

where m is body mass including equipment, ${\text{F}}_{{\text{pole}}_{⊥}}$ is pole force perpendicular to treadmill belt, and ${\text{v}}_{{\text{ski}}_{∥}}$ is the velocity of the roller skis parallel to treadmill belt [e.g., 16].

E_body_ includes energy related to movements in goal-direction (speed fluctuation) as well as energy mainly related to the repetitive heightening-and-lowering of the body throughout the DP cycle. An attempt was made to estimate the ‘internal’ energy changes related to these up-and-down body movements perpendicular to the surface, and its relation to fluctuation in instantaneous pole power. This was done unambiguously in ergometer DP [11], where body energy changes were shown to be largely out-of-phase with instantaneous poling power, indicating the transfer of body energy to pole power as the mechanism allowing the lower extremity to contribute (indirectly) in generation of pole power. In the current study, the power associated with movements in goal-direction (P_∥_) was estimated according to Dahl et al. [16]:

$${\text{P}}_{∥}={\text{v}}_{{\text{CoM}}_{∥}}(\text{mg}\;\sin\;\alpha +(\text{mg}\;\text{cos}\;\text{a}-{\text{F}}_{{\text{pole}}_{⊥}})\mu +{\text{ma}}_{{\text{CoM}}_{∥}})$$ (5)

with ${\text{v}}_{{\text{CoM}}_{∥}}$ and ${\text{a}}_{{\text{CoM}}_{∥}}$ the velocity and acceleration of CoM in goal-direction (i.e., parallel to the treadmill belt), respectively. Subtracting P_∥_ from P_o_ was defined as the rate of change of body energy associated with body movements perpendicular to the treadmill belt (${\text{dE}}_{{\text{body}}_{⊥}}/\text{dt}$), that is, energy mainly related to the repetitive body lowering and heightening throughout the DP cycle

$${\text{dE}}_{{\text{body}}_{⊥}}/{\text{dt}=\text{P}}_{\text{o}}-{\text{P}}_{∥}$$ (6)

Instantaneous pole power (P_pole_) was calculated as:

$${\text{P}}_{\text{pole}}={\text{F}}_{\text{pole}}\;{\text{V}}_{\text{CoM}}\;\cos\;\beta$$ (7)

where → F_pole_ is the pole force vector (which direction was determined from the pole markers), V_CoM_ is the CoM velocity vector (relative to treadmill belt speed), and *β* is the angle between these two vectors [e.g., 16, 23]. Averaged over a cycle, P_pole_ should be equal to cycle mean P_o_.

Inverse dynamics [24] were used to calculate net shoulder and elbow joint moments, and joint power was calculated as the dot product of joint moment and joint angular velocity. The sum of elbow and shoulder power was defined as arm power (P_arm_). Because of considerable within-trunk movements and a lack of measurements of ski reaction forces and its point of application, inverse dynamics was only performed for the upper extremity. The difference between P_o_ and P_arm_ was considered as power associated with trunk and leg movements (trunk+leg, P_T+L_), in a similar way as in [8, 25].

In order to examine how the power contribution from P_arm_ and P_T+L_ depends on speed, a method similar to that which has been used previously was adopted [8, 26, 27]. The power curves of P_arm,_ and P_T+L_ were integrated over the duration of the cycle and the poling and swing periods to yield the work done in the respective phases. These work values were divided by cycle time, poling time, and swing time to yield average power ($\bar{\text{P}}$). Furthermore, P_arm_ and P_T+L_ were time integrated over their respective positive and negative periods only, independently for the poling and swing phases. The sum of all positive and negative work values for the poling and swing phase was then divided by poling time and swing time, respectively. This yielded average positive (${\bar{\text{P}}}^{+}{}_{\text{UB}}$ and ${\bar{\text{P}}}^{+}{}_{\text{TLE}}$) and negative (${\bar{\text{P}}}^{-}{}_{\text{UB}}$ and ${\bar{\text{P}}}^{-}{}_{\text{TLE}}$) arm and trunk+leg power in the poling and swing phases.

All data were time normalized for each participant for each cycle and averaged over ~20 cycles for each condition, and group mean ± 95% confidence interval (CI) curves were obtained by averaging across all participants.

### Statistical analysis

All data were checked for normality by visual inspection of normal Q-Q plots and histograms and are presented as means ± 95% CI. One-way repeated measures ANOVAs were performed to analyse main effects of speed and *post hoc* analyses (Fischer least significant difference) were used to evaluate differences between speeds. Statistical significance was set at *P*<0.05 and all statistical tests were performed using SPSS version 24 (IBM Inc., Armonk, NY, USA) and Microsoft Excel version 14.0.7190.5000 (Office 2016, Microsoft Corporation, Redmond, WA, USA).

## Results

### Basic cycle characteristics

Table 1 shows the basic cycle characteristics. Cycle average P_o_ and P_pole_ were quite similar, with the differences indicating measurement accuracy. Cycle length and rate increased with speed, while both absolute and relative poling time decreased. Swing time was less affected by speed.

**Table 1:** Variables associated with double-poling treadmill roller-skiing on 1% inclination at increasing speeds.

Variables	Speed				
	Low	Mod	High		
	15 km∙h-1	21 km∙h-1	27 km∙h-1	*P*	$\eta_{\text{p}}^{\text{2}}$
P_pole_ (W)	89 ± 5^bc^	136 ± 7^ac^	182 ± 8^ab^	<0.001	0.99
P_o_ (W)	99 ± 5^bc^	138 ± 7^ac^	176 ± 8^ab^	<0.001	0.99
Cycle rate (Hz)	0.72 ± 0.02^bc^	0.75 ± 0.02^ac^	0.81 ± 0.03^ab^	<0.001	0.75
Cycle length (m)	5.76 ± 0.18^bc^	7.74 ± 0.24^ac^	9.21 ± 0.41^ab^	<0.001	0.97
Poling time (s)	0.45 ± 0.02^bc^	0.35 ± 0.02^ac^	0.29 ± 0.01^ab^	<0.001	0.97
Relative poling time (%)	32 ± 1^bc^	26 ± 1^ac^	23 ± 1^ab^	<0.001	0.93
Swing time (s)	0.94 ± 0.04^b^	0.99 ± 0.04^ac^	0.95 ± 0.05^b^	0.034	0.23
Max CoM height (m)	1.10 ± 0.02^bc^	1.12 ± 0.02^ac^	1.15 ± 0.02^ab^	<0.001	0.87
Perpendicular CoM displacement (m)	0.14 ± 0.01^bc^	0.19 ± 0.01^ac^	0.26 ± 0.02^ab^	<0.001	0.96

Values are mean ± 95% CI, *P*-values and $\eta_{\text{p}}^{2}$ for the repeated measures ANOVA [N = 14]. a, b, and c indicate significant differences from low, moderate, and high speeds, respectively (*P*<0.05).

P_pole_, pole power; P_o_, net power output; CoM, centre of mass.

### Mechanical energy fluctuations

**Fig 1:**
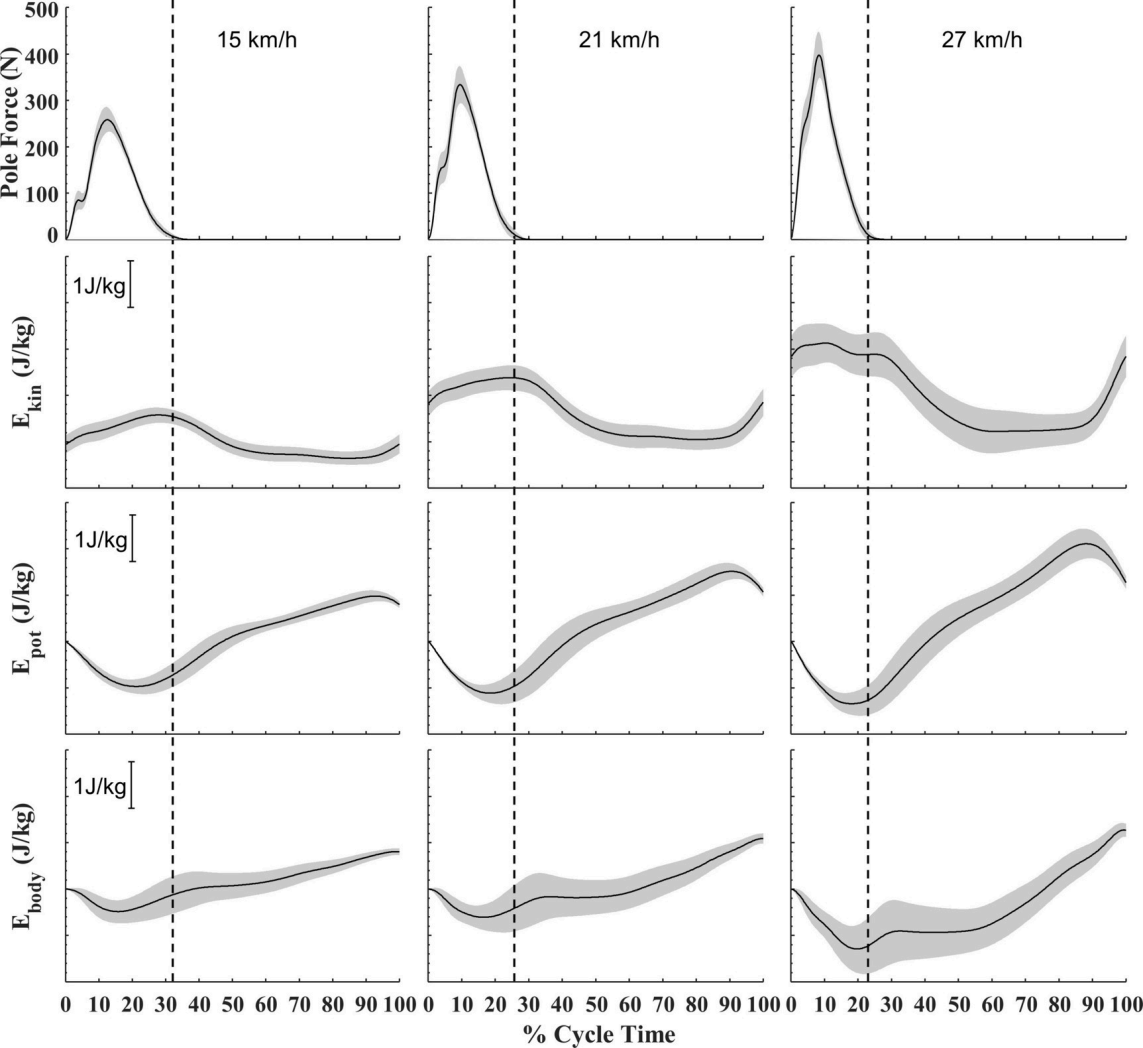
Pole force and mechanical energy during double poling at increasing speeds on a 1% inclined treadmill. Plots are the average of all subjects [N = 14] with 95% CI indicated with shaded areas. Dashed vertical lines represent end of poling phase (mean of all subjects). Energy are shown normalized to body weight, with unit indicated by vertical bars. E_pot_ and E_body_ are presented relative to the energy levels at the start of a cycle.

Fig 1 shows fluctuations in E_kin_, E_pot_ and E_body_ together with F_pole_ at the three speeds. F_pole_ rapidly increased from onset of poling and peak F_pole_ was reached slightly before halfway through the poling phase. E_body_ was changing throughout the cycle, decreasing during the poling phase and increasing during the swing phase. Towards the end of the swing phase, E_pot_ started to decrease (body lowering). At the faster speeds, this rate of decrease in E_pot_ became higher, leading to an increase in E_kin_ towards the end of swing (body accelerating downwards). E_pot_ continued to decrease into the poling phase, but started to increase slightly before poling was ended. Both during poling and swing E_kin_ and E_pot_ tended to fluctuate out-of-phase; however, the magnitude of the decrease in E_pot_ was somewhat larger than the increase in E_kin_ during poling, leading to a net decrease in E_body_ for the majority of the poling phase, especially at the highest speed. E_kin_ generally increased during poling (mainly because of forward acceleration). Throughout the swing phase, E_kin_ mostly decreased (speed lost to rolling resistance) while E_pot_ substantially increased (perpendicular body heightening as well as rising along the slope). The amount of perpendicular displacement of CoM increased with speed, mostly because the minimal height of CoM during the poling phase decreased (Table 1).

### Mechanical power

**Fig 2:**
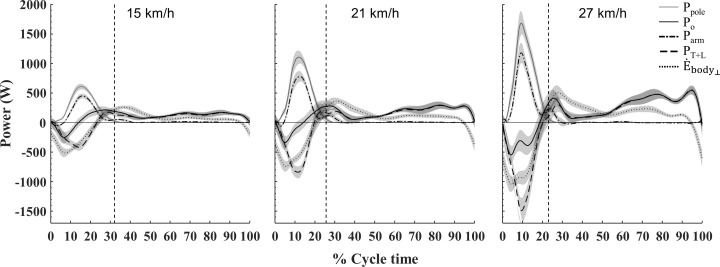
Mechanical power during double poling at increasing speeds on a 1% inclined treadmill. Plots are the average of all subjects [N = 14] with 95% CI indicated with shaded areas. Dashed vertical lines represent end of poling phase (mean of all subjects).

Fig 2 shows fluctuations in mechanical powers. At all intensities, P_pole_ and ${\dot{\text{E}}}_{{\text{body}}_{⊥}}$ generally fluctuated out-of-phase during the poling phase. The peak in negative ${\dot{\text{E}}}_{{\text{body}}_{⊥}}$ (decreasing ‘internal’ body energy) occurred slightly before the peak in P_pole_ (pole propulsion power). Most power characteristics regarding pattern during the cycle seemed unchanged with speed. However, P_o_ showed a fundamental change with increasing speed. At low speed, P_o_ shifted from negative during the first part of the poling phase to positive at the second part. At high speed however, P_o_ remained negative for the majority of the poling phase.

**Fig 3:**
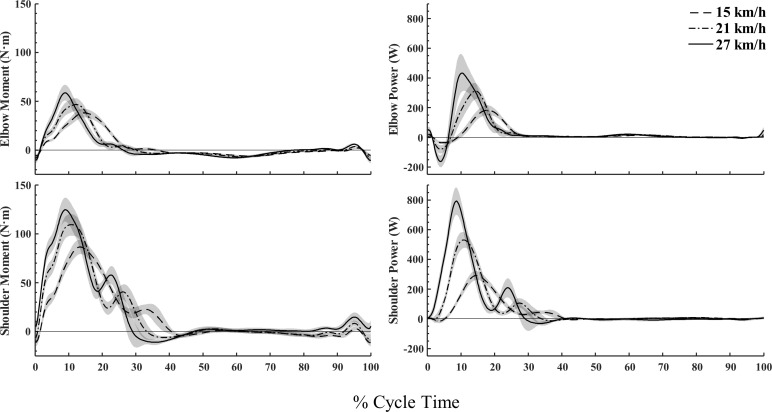
Net joint moment and joint power at the elbow and shoulder during double poling at increasing speeds on a 1% inclined treadmill. Plots are the average of all subjects [N = 14] with 95% CI indicated with shaded areas.

P_arm_ rapidly increased from onset of poling and followed the same pattern as P_pole_. P_T+L_ was negative for the majority of the poling phase (following a similar pattern as ${\dot{\text{E}}}_{{\text{body}}_{⊥}}$), before becoming positive towards the end of poling and remained positive throughout swing. Thus, both considerable power generation (arm) and absorption (trunk+leg) occurred simultaneously during the poling phase. More specific upper-extremity dynamics are shown in Fig 3. During poling, an extensor moment was dominating both the elbow and shoulder with the shoulder moment being of a larger magnitude. From onset of poling, positive shoulder power rapidly increased, especially at higher intensities. The elbow displayed a brief period of negative power during the first part of the poling phase (elbow flexion), before positive elbow power rapidly increased (elbow extension). Peak positive shoulder power was reached slightly before peak positive elbow power.

Table 2 shows average positive and negative power for the whole cycle and for poling and swing phases. Over the cycle, both arm and trunk+leg power increased with speed. However, the relative contribution changed only slightly; relative arm power increased from 63% at low speed to 66% at moderate and high speed (p = 0.016). During poling, positive arm power increased considerably with speed, as did negative trunk+leg power. During swing, positive trunk+leg power increased. The work done by the trunk+leg during the swing phase amounted to 113 ± 15 J, 180 ± 19 J, and 275 ± 20 J, while the energy absorbed by the trunk+leg during the poling phase amounted to -81 ± 11 J, -131 ± 14 J, and -206 ± 17 J.

**Table 2:** Mechanical power (W) associated with double-poling treadmill roller-skiing on 1% inclination at increasing speeds.

Variables	Speed				
	Low	Mod	High		
	15 km∙h^-1^	21 km∙h^-1^	27 km∙h^-1^	*P*	$\eta_{\text{p}}^{2}$
**Cycle**					
${\bar{\text{P}}}_{\text{arm}}$ (W)	62 ± 4^bc^	91 ± 6^ac^	115 ± 8^ab^	<0.001	0.97
${\bar{\text{P}}}_{\text{T+L}}$ (W)	37 ± 4^bc^	47 ± 7^ac^	61 ± 8^ab^	<0.001	0.90
${\bar{\text{P}}}_{\text{arm}}$ (%)	63 ± 3^bc^	66 ± 4^a^	66 ± 4^a^	0.016	0.27
${\bar{\text{P}}}_{\text{T+L}}$ (%)	37 ± 3^bc^	34 ± 4^a^	34 ± 4^a^	***-***	***-***
**Poling**					
${\bar{\text{P}}}^{+}{}_{\text{arm}}$ (W)	176 ± 15^bc^	327 ± 31^ac^	471 ± 43^ab^	<0.001	0.97
${\bar{\text{P}}}^{+}{}_{\text{T+L}}$ (W)	46 ± 9^bc^	51 ± 15^ac^	30 ± 17^ab^	<0.001	0.30
${\bar{\text{P}}}^{-}{}_{\text{arm}}$ (W)	-6 ± 2^c^	-6 ± 2^c^	-1 ± 1^a^	<0.001	0.69
${\bar{\text{P}}}^{-}{}_{\text{T+L}}$ (W)	-182 ± 25^bc^	-383 ± 46^ac^	-727 ± 87^ab^	<0.001	0.93
**Swing**					
${\bar{\text{P}}}^{+}{}_{\text{arm}}$ (W)	12 ± 1^bc^	15 ± 1^ac^	17 ± 1^ab^	<0.01	0.50
${\bar{\text{P}}}^{+}{}_{\text{T+L}}$ (W)	122 ± 19^bc^	184 ± 23^ac^	293 ± 28^ab^	<0.001	0.95
${\bar{\text{P}}}^{-}{}_{\text{arm}}$ (W)	-1 ± 0^c^	-2 ± 1^c^	-7 ± 2^a^	<0.001	0.81
${\bar{\text{P}}}^{-}{}_{\text{T+L}}$ (W)	-1 ± 1^c^	-2 ± 2^c^	-6 ± 4^a^	0.013	0.35

Values are mean ± 95% CI, *P*-values and $\eta_{\text{p}}^{2}$ for the repeated measures ANOVA [N = 14]. a, b, and c indicate significant differences from low, moderate, and high speeds, respectively (*P*<0.05).

P_arm_, arm power, P_T+L_, trunk+leg power.

## Discussion

The present study examined the effect of increasing speed on fluctuations in body mechanical energy and propulsion mechanics during roller-skiing DP on level terrain. As hypothesized, the perpendicular heightening and lowering of the body and related mechanical energy fluctuations became more pronounced at faster speeds. As speed increased, the body was lowered more and faster during the poling phase and thus heightening and repositioning of the body (mainly during the swing phase) also increased and occurred faster. This was reflected in an increased fluctuation of E_pot_ over the cycle. The body and pole repositioning during the swing phase leads to an increase in body mechanical energy, delivered mainly by trunk+leg work. During the poling phase, the mechanical (potential) body energy gained during swing is released, a predominant amount of which seems to be absorbed again by the trunk+leg. Thus, pole propulsion power is generated to a great extent by the arms (~63–66%) while a significant amount of the body mechanical energy released, that is, energy generated by predominantly trunk+leg actions in the previous swing phase, makes up the remaining part (~34–37%). Accordingly, the fluctuation in internal body energy and (external) pole power during the poling phase are largely out-of-phase in roller-skiing DP, and comparable to ergometer DP [11]. Overall, the main effect of speed is an enhancement of most abovementioned processes, with one exception: The change of total (joint) power during the poling phase which shows a negative-positive pattern at low and moderate speeds, changing to an almost continuously negative value at high speed. That is, at low and moderate speeds, the power delivery by the arms and absorption by the trunk+legs are more in balance than at high speed, in which trunk+leg power absorption exceeds the delivery in the arms by far.

### Mechanical power contribution

Averaged over the whole cycle, arm power contribution was 63% at low speed (work rate 98 W) and 66% at high speed (work rate 176 W). This is quite different from what was found in ergometer DP [8] at comparable work rates, where the arm power contribution decreased from 51% at low-intensity DP (work rate 116 W), to 47% (work rate 166 W) and further to 43% (work rate 214 W). The values obtained here in level roller-skiing DP are thus more similar to those reported recently for uphill roller-skiing DP [9]. In uphill DP [9], arm power contribution was 63% at moderate (5%) and 54% at steep incline (12%), and unaffected by increasing speeds at both those inclines [9]. The finding of little or no effect of intensity/speed on these power contributions during roller-skiing DP are in contrast to several other studies which have shown, although using measures other than joint power, that the involvement of the trunk+leg increases when DP intensity or speed is increased [4, 7, 14, 28, 29]. It should be noted that the legs and trunk, and even different parts of the legs (for example hip versus knee) may have responded differently by the increase in speed than what is possible to deduce here, since trunk and leg power is considered together. That is, the summed lower extremity joint power (ankle, knee, hip) contribution may be different than what is found here. The effect of speed (or intensity) at the lower extremity joint level as well as trunk functioning is indeed interesting and should be studied further.

One reason for not finding an increase in trunk+leg contribution here in level roller-skiing DP, might be related to the amount of negative trunk+leg power during the poling phase. Compared to ergometer DP [8, 11], trunk+leg work performed during the swing phase are more in balance with trunk+leg energy absorption during poling. The finding of an increasing amount of negative trunk+leg power at faster speeds in the present study are similar to the findings in moderate uphill DP [9]. In that study, the magnitude of negative trunk+leg power increased substantially at faster speeds also at a 5% inclined treadmill, but not so much at a 12% inclined treadmill. An explanation given there [9] was the requirement of the legs to support more of body weight at moderate inclines, while the poles must take up more of this weight at steeper inclines. This is especially apparent during the first part of the poling phase, in which the body is rapidly and actively lowered. The flatter the surface, the more the legs apparently must counteract this downward momentum [6]. That is, in level DP, more of the decreasing body energy must seemingly be absorbed by the legs (and trunk), whereas on steeper inclines more of this energy (although less in absolute terms) can be directly transferred into pole power [9].

In addition, a related reason is likely the decrease in poling time, which becomes shorter and shorter at faster DP speeds (e.g., <0.25 s). The only way for the skier to be able to generate enough pole force and power over such short time periods seems to be by relying increasingly more on whole-body movements, i.e. using body mass to a greater extent to increase pole force [4, 5, 8, 28]. This leads to the more jumping-like DP technique with rapid up-and-down and forward leaning body movements. However, a large part of the increased body energy (gained from trunk+leg power generation during swing) is apparently absorbed again by the trunk+leg during the following poling phase, and so these enhanced up-and-down movements does not show up as an increase in trunk+leg power contribution. It may be, however, that this strategy is essential for effective arm and pole power generation at these faster speeds by allowing for high pole forces. This technical strategy positions the body and poles so that much arm power can be generated in a short time period.

Moreover, it is questionable whether the finding of positive-negative trunk+leg power is effective in terms of metabolic cost. Although likely a prerequisite for effective generation of pole force and power, it can still be hypothesized that these findings may partly explain some of the decrease in efficiency seen in level DP at faster speeds [10]. However, at some point, an athlete must simply use any strategy possible to generate the power required to obtain or maintain a certain speed, even though that strategy may lower efficiency compared to the technical strategy used on slower speeds.

Regarding efficiency or energetic cost of DP, Zoppirolli et al. [30] found that better skiers have a lower energetic cost of DP than less good skiers, which was related to less vertical displacement of the CoM throughout the cycle. At first sight, that finding [30] may not seem to agree with the main mechanisms of DP propulsion mechanics described here and previously [4, 5, 10, 11], that is, vertical CoM fluctuations and active leg work is an essential part of DP technique and becomes more pronounced at faster speeds. However, when relating the large amount of negative trunk+leg power found here (and on a moderate incline [9]), the findings by Zoppirolli et al. [30] are not necessarily in contradiction to our findings, but rather emphasize that better skiers might have a better technique in which a required and more optimal amount of vertical CoM oscillation is included, whereby possible energy waste may be less. Since one can never store and reutilise the full amount of decreasing energy in a countermovement-like action, lowering energy loss is important for energy effectiveness. The less vertical CoM movement of the better skiers found in that study [30] was mainly due to less CoM lowering during poling, indicating less need for energy absorption by trunk+legs. Still, completely avoiding vertical CoM movement in DP seem to contradict a main propulsion mechanism, i.e., allowing the legs to contribute in the generation of pole force and power [5, 6, 11]. Furthermore, as shown in for example walking and running, minimizing vertical CoM movements may in fact increase the metabolic cost of transport [31–33]. Apparently, future studies should examine further the role of vertical CoM oscillation and the relation to DP skiing economy and efficiency, especially whether less vertical CoM movement of better skiers is related to less negative trunk+leg power and whether this decreases energetic cost at different speeds and/or inclines. Such information would be important for practice, that is cross-country skiers and coaches.

### Mechanical energy fluctuations

The patterns of mechanical energy fluctuations found here are similar to those shown by Pellegrini et al. [12]. Based on the out-of-phase fluctuations in E_kin_ and E_pot_, they [12] argued that DP resembles a pendular style of locomotion as seen in for example walking. In walking [23, 34–36], the CoM gains height and loses speed during the first part of single support as the CoM vaults over the rigid stance limb, converting E_kin_ into E_pot_. During the second half of single support, the opposite occurs. This direct exchange between E_kin_ and E_pot_ during each single stance (each pendulum phase), traditionally considered a mostly passive mechanism that lowers the requirement for active muscle work, offers evidence for the pendulum analogy applied to single stance in walking [35, 37], during which very little active joint (muscle) work is done [27]. In light of this, it seems as if DP does not resemble pendulum-like behaviour, except maybe during the transition period between swing to poling phases. Despite the out-of-phase fluctuation in E_pot_ and E_kin_, for example during the poling phase, considerable active (upper body) muscle work is done simultaneously, indicating that the system may not be considered passive. Thus, inferring the poling phase as a motion where the skier ‘vaults’ over the poles as a passive mechanism (little muscle work) does not completely comply to the analogies used during the single stance phase in walking [e.g., 27], especially since the body is (actively) lowered during most of poling phase. A much used measure for quantifying the possible amount of exchange (recovery) between energy associated with forward movements and energy associated with vertical movements is R_cycle_ (see [12, 35]). We found R_cycle_ of values of 55±8%, 55±6%, and 50±7%, which are somewhat higher but comparable to the 45% reported previously in DP at 3.5% incline and 14 kmh [12] and also comparable to those found in level walking at comfortable speeds (50–60% [38]). We still caution the applicability of this concept in locomotion such as DP, mainly since it is not clear what the pendulum mechanism itself is (compared to walking, where CoM valuting over rigid stance leg applies) and because considerable work is done over the cycle as well as instantaneously at the same time points in which recovery is high [37].

A considerable part of the generated pole power originated not from instantaneous muscle work, but from a transfer of part (~34–37%) of the body energy [39]. During the swing phase, the increase in E_pot_ is mainly due to active trunk+leg work, and it is unclear (given DP movement characteristics) how this increase in E_pot_ could originate from a (passive) conversion of E_kin_ into E_pot_, as in for example walking. Furthermore, the decrease and increase in E_pot_ and related positive-negative trunk+leg power suggest that reutilisation of energy in bouncing-like movements may occur, meaning that bouncing-ball-like rather than pendulum-like behaviour may apply to level DP. These bouncing-like movements became more apparent at faster speeds, reflected in the large increase in negative trunk+leg power during the poling phase, and also coincided with a decrease in R_cycle_. Taken together, although the effect of speed on relative arm (and trunk+leg) power contribution was not large at the speeds studied here, the DP technique was affected in the sense that both negative (legs) and positive (arm) power became much larger. Accordingly, the findings of this study suggest that DP increasingly more resembles bouncing-like movements at faster speeds.

## Conclusion

Increasing speed in roller-skiing DP on level terrain leads to increased and faster body lowering and heightening during the poling and the swing phases. Thus, E_pot_ increases during swing due to trunk+leg power generation. During the poling phase, a part of the decreasing body (potential) energy is delivered to pole propulsion power. Over the whole cycle, arm power contribution was 63% at low and 66% at moderate and high speeds, the rest originating from trunk+leg power. Hence, although the involvement of the trunk and legs increase at faster DP speeds (more and faster body heightening-and-lowering), there was no increase in trunk+leg power contribution at the speeds considered here. A reason for this may be the large increase in negative trunk+leg power during the poling phase at faster speeds. Future studies should further examine whether the large increase in trunk+leg power generation and absorption at faster speeds may be related to cross-country skiers’ performance level and possible to a decrease in energetic cost of DP. Although the relative trunk+leg contribution did not increase, the technical strategy (enhanced body heightening and lowering) did, leading to the large increase in trunk+leg both power generation and absorption. This may still be considered to be a prerequisite for effective generation of pole force and pole power during high-speed DP.

## Supporting information

S1 File (XLSX)
